# An Exceptionally Active and Highly Selective Perchlorate Transporter Containing a Trimesic Amide Scaffold

**DOI:** 10.3390/molecules29051118

**Published:** 2024-03-01

**Authors:** Shaowen Deng, Zhongyan Li, Lin Yuan, Huaqiang Zeng

**Affiliations:** 1 College of Chemistry and Bioengineering, Hunan University of Science and Engineering, Yongzhou 425100, China; shaowen_deng@163.com; 2 College of Chemistry, Fuzhou University, Fuzhou 350116, China; lizhongyandongdong@126.com

**Keywords:** trimesic amide, perchlorate anion, transmembrane, anion transporter

## Abstract

We report here a series of alkyl group-modified trimesic amide molecules (**TA**s) with excellent anion transport activities. Among them, **TA6,** with the highest ion transport activity and excellent selectivity, efficiently transports anions across the membrane in the order of ClO_4_^−^ > I^−^ > NO_3_^−^ > Br^−^ > Cl^−^, with an *EC*_50_ value as low as 17.6 nM (0.022 mol% relative to lipid molecules) for ClO_4_^−^, which outperforms other anions by 5- to 22-folds and manifests as the best perchlorate transporter ever reported.

## 1. Introduction

Intracellular life activities are closely related to specific ion concentrations, which are regulated by channel proteins on the cell membrane. The imbalance of specific ions can lead to a series of channel diseases, such as cystic fibrosis (Cl^−^) [1,2], iodine deficiency, paralysis (Na^+^) [3,4], epilepsy (K^+^) [5,6] and malignant hyperthermia (Ca^2+^) [7,8,9,10]. Therefore, the study of ion transport by membrane channel proteins and their synthetic analogs is of great significance to human health. Given the complex structure of natural membrane proteins, it is still very challenging to design and synthesize artificial ion channels [11,12,13,14,15,16,17,18,19,20,21,22,23,24,25], which can achieve excellent transport functions seen in the protein channels.

At present, most of the artificially designed and synthesized ion channel molecules carry central macrocyclic units, tubular cavities or one-dimensionally aligned ion-binding/transporting units [26,27,28,29,30]. Inspired by the naturally occurring pore-forming peptides [31,32,33,34] and toxins [35], α-helix bundles [35,36,37,38,39] and protein channels [40,41], which relay on side chain–side chain interactions to assemble into transmembrane pores for cell killing or controlling substance exchanges across the membrane, one recently emerging strategy also made similar uses of such noncovalent interactions to drive the channel formation for inducing transmembrane anion transport [42,43,44,45,46].

**Figure 1:**
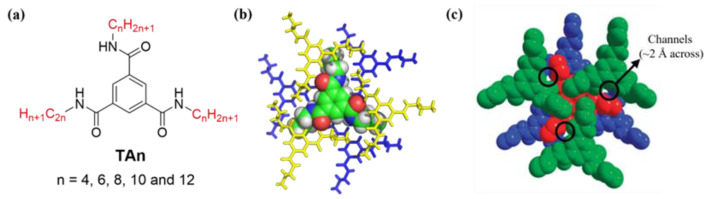
(**a**) Molecular design and chemical structures of **TAn** (n = 4, 6, 8, 10 and 12) studied in this work. (**b**) Crystal structure of **TA3**, showing that every **TA** molecule is linked to six adjacent molecules through intermolecular H-bonds with three molecules above and the other three below it; (**c**) **TA3′**s molecular packing in solid state to generate noticeable pores measuring 2 Å across [56].

Trimesic amides (**TA**s) found many applications in such as small-molecule gelators [47], low-molecular-weight organogelators [48], polyvalent inhibitors [49], artificial receptors [50], dendrimers [51], porous coordination polymers [52], supramolecular polymers [53], organic diodes [54], chiral amplification [55] and thermoplastic elastomer [56]. The three amide groups in **TA** are aligned in such a way that there is a tilting angle relative to the central benzene ring. While this angle is highly dependent on the peripheral substituents, it is responsible for generating intermolecular stacking among **TA**s via intermolecular H-bonds and the π-π stacking forces. In a particular case such as **TA3** (Figure 1a,b), a triple helical network of intermolecular H-bonds cross-links six adjacent **TA3** molecules, and the concurrent side chain–side chain associations produce sizeable channels measuring 2 Å across (Figure 1c) [57].

We speculated that such a small hydrophobic pore may become sufficiently or transiently larger to achieve specific and efficient anion transport by fine-tuning the length of the appended alkyl chains or when the molecules are embedded within the lipid bilayer membrane. To confirm this hypothesis, we designed and synthesized five **TA** molecules (**TAn**, n = 4–12, Figure 1a).

## 2. Results and Discussion

**Figure 2:**
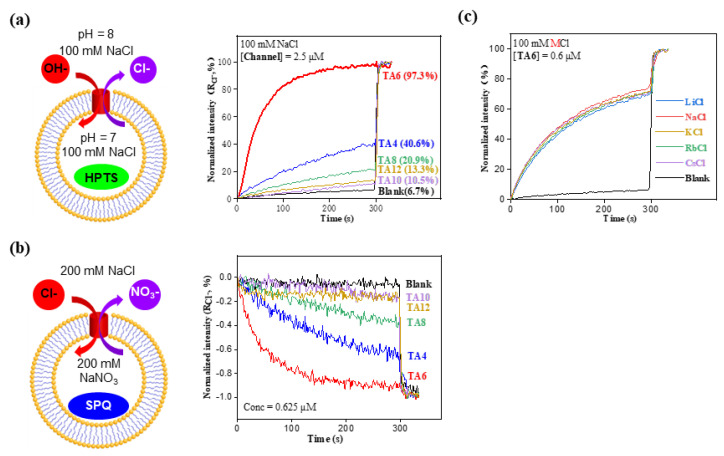
(**a**) Schematic diagram of the HPTS assay for evaluating ion transport activities of **TAn** at 2.5 μM; here, R_Cl_^−^ = (I_Cl_^−^ − I_0_)/(I_Triton_ − I_0_), whereas I_Cl_^−^ and I_0_ are the ratiometric values of I_460_/I_403_ at t = 300 s before addition of triton, and I_Triton_ is the ratiometric value of I_460_/I_403_ at t = 300 s right after addition of triton. (**b**) Chloride-sensitive SPQ assay confirming that **TA**s can transport chloride anions. (**c**) Ion transport activities by varying the extravesicular MCl salts, confirming that **TA**s lack the ability to transport cations.

To evaluate the ion transport activities of these five **TA** transporters, we performed the HPTS-based LUV assay, with both intra- and extravesicular regions filled with 100 mM NaCl and the same type of HEPES buffer (Figure 2a), with a pH gradient of 7 to 8 across the membrane. In this method, one of the four ion exchange modes (Na^+^/H^+^ antiport, Na^+^/OH^−^ symport, Cl^−^/OH^−^ antiport or Cl^−^/H^+^ symport) may occur during the ion transport process, inevitably resulting in an increase in the pH inside the LUV and consequently increasing the fluorescence intensity of the pH-sensitive HPTS dye. At the same transporter concentration, the changes in fluorescence intensity over time can be used to compare the relative ion transport capacity of transporters. Using this assay, at 2.5 μM, hexyl group-containing **TA6** showed the highest ion transport activity of 97%, followed by **TA4** > **TA8** > **TA12** > **TA10** (Figure 2a).

Although we are not exactly sure why **TA6** is the best transporter, given that the pores formed by **TA** molecules are generated via side chain–side chain interactions, these alkyl side chains should play two important roles. That is, certain parts of the chain are engaged in the pore formation, with certain parts of the chain responsible for generating sufficiently large side chain–side chain interaction forces to maintain the pore. Therefore, we believe an intermediate chain length, as in **TA6,** enables both roles to be fulfilled well. While the shorter chain length, as in **TA4**, cannot produce sufficiently large side chain–side chain interaction forces to maintain the pore, the longer chains in **TA8**–**TA12** might favor side chain–side chain interactions over the pore formation.

As hypothesized, **TA** molecules may associate with each other via side chain–side chain interactions to generate the pores for ion transport. In this pore-forming pathway, the interior of the pore is going to be very hydrophobic, most likely favoring the transport of anions rather than cations. To elucidate which types of ions are transported by **TA** molecules, the chloride-sensitive SPQ assay was conducted first to assess the chloride transport capacity of **T**A transporters (Figure 2b). The principle behind this assay is that the fluorescence intensity of SPQ dye is quenched with an increasing concentration of chloride anions. If **TA** molecules transport anions, an exchange of NO_3_^−^ and Cl^−^ will take place, leading to decreased intensities of SPQ dyes. Based on the extent of the fluorescence intensities of SPQ, we observed the chloride transport activities of the five transporters in the order of **TA6** > **TA4** > **TA8** > **TA12** > **TA10** (Figure 2b), a trend that is in excellent consistency with the above activity order obtained using the HPTS assay (Figure 2b).

To rule out the possibility of cation transport, the ion transport activities were also measured by varying the extravesicular MCl salts (M = Li, Na, K, Rb and Cs, Figure 2c). If **TA** molecules transport cations by varying the M^+^ ions in the extravesicular region, one expects to see significant differences in ion transport activity. Our results, however, reveal insignificant differences in transport activity, indicating that the metal ions were not transported by **TA6** or the corresponding transport activities were almost the same.

**Figure 3:**
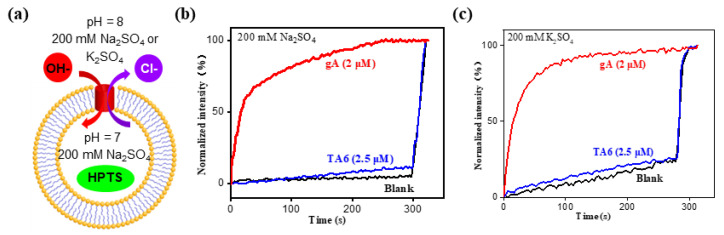
(**a**) The sulfate-containing HPTS assay, which confirms **TA** molecules do not transport (**b**) either Na^+^ or (**c**) K^+^ ions.

Further, using the LUV assay containing 200 mM Na_2_SO_4_ (Figure 3a), the change in fluorescence intensity of **TA6** at 2.5 μM was nearly identical to that of the background signal, demonstrating that **TA6** does not mediate transports of Na^+^ ions, protons, OH^−^ and SO_4_^2−^ anions. In contrast, the fluorescence intensity increased by almost 100% when Na^+^/proton-transporting gramicidin A (gA) was present at 2 μM (Figure 3b). Similar results were obtained when the extravesicular Na_2_SO_4_ was changed to K_2_SO_4_ (Figure 3c). Taken together with the data from Figure 2c, these data clearly show that transporters **TAn** transport anions rather than cations or protons.

These above comparative results collectively establish chloride ions as the transportable ions by **TA** transporters, thereby suggesting the ion exchange mode during the **TA**-mediated ion transport process to be either Cl^−^/OH^−^ antiport or Cl^−^/H^+^ symport. Considering the central highly hydrophobic region of the membrane favors OH^−^ over H^+^, the hydrated OH^−^ anions should be more capable of passing the membrane than hydrated H^+^ ions. In other words, Cl^−^/OH^−^ antiport exchange is more likely, with **TA**-mediated influx of the chloride ions compensated by the passive efflux of OH^−^ to reach a charge neutrality condition of the system.

**Figure 4:**
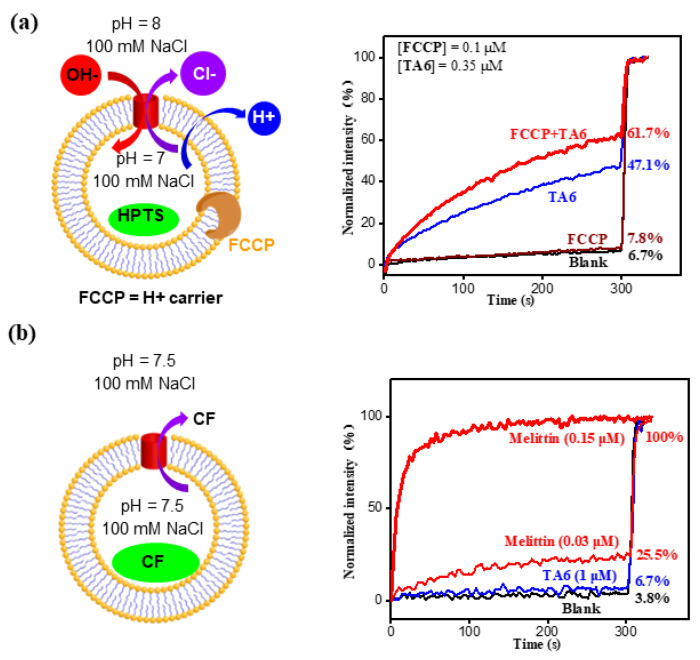
(**a**) FCCP-based LUV assay for assessing the relative transport rates between OH^−^ and Cl^−^. (**b**) CF dye assay to reveal membrane integrity in the presence of **TA** molecules or **TA** molecules does not generate pores larger than 1 nm.

To differentiate the transport rate between Cl^−^ and OH^−^ anions, a potent H^+^ carrier, **FCCP** (carbonyl cyanide 4-(trifluoromethoxy) phenylhydrazone), was employed (Figure 4a). For this assay, if the transport rate of Cl^−^ is faster than that of OH^−^, the build-up of OH^−^ over time will be quickly dissipated by the FCCP-mediated efflux of protons, resulting in considerably larger changes in fluorescence intensity. What was seen in Figure 4b is in great accord with such expectation, implying that the transport rate of Cl^−^ is faster than OH^−^ anions.

Applying 5(6)-carboxyfluorescein (CF) molecules that have a dimension of <1.0 nm in size and that self-quench at high concentration, the CF assay, having intravesicular region filled with CF at 50 mM, was carried out (Figure 4b). We found that the lysosomal melittin, which forms transmembrane pores of >1 nm, led to 25.5% and 100% outflow of CF molecules at 0.03 and 0.15 μM, respectively (Figure 4b). Even at a concentration of 1 μM, **TA6**, however, only produced a negligible CF efflux of 2.9%. These findings clearly support that (1) the integrity of the membrane is well maintained in the presence of a high concentration of **TA6,** and (2) **TA6** does not generate pores larger than 1 nm.

**Figure 5:**
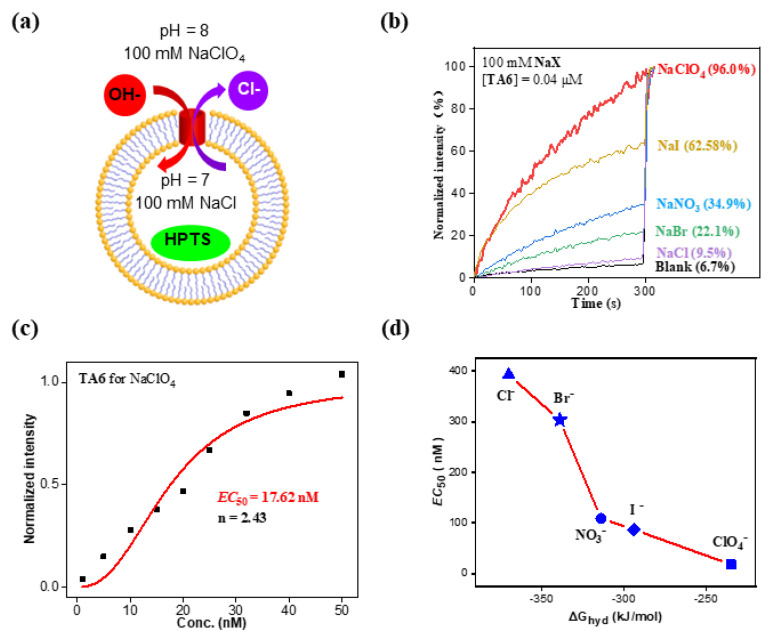
(**a**) and (**b**) illustrate **TA6**-mediated anion selectivity obtained by varying extravesicular NaX salts. (**c**) *EC*_50_ value determined for **TA6**-mediated perchlorate transport and (**d**) correlation between *EC*_50_ values and the hydration energies of anions.

To determine the anion selectivity of the best transporter **TA6**, both the intravesicular and extravesicular NaX salts were systematically varied to include five types of anions (Figure 5a). It was found that **TA6** exhibits a very significant anion selectivity in the order of ClO_4_^−^ > I^−^ > NO_3_^−^ > Br^−^ > Cl^−^ at a concentration of 0.04 μM (Figure 5b). More quantitative results in anion selectivity were obtained by determining the *EC*_50_ values at which the transporters reach 50% ion transport activity. As shown in Figure 5c,d and Supplementary Materials Figures S1–S5, the *EC*_50_ values of different anions for **TA6** were 17.6 nM (0.022 mol% relative to lipids) for ClO_4_^−^, 86.6 nM for I^−^, 108.6 nM for NO_3_^−^, 303.3 nM for Br^−^ and 392.6 nM for Cl^−^. Based on these *EC*_50_ values, **TA6** transports ClO_4_^−^ 5–22 times better than the other four anions. Interestingly, the **TA6**-mediated anion transport activities increase with decreased hydration energy (Figure 5d).

Lastly, we have attempted but failed to record the single-channel currents. One possible reason is that pores formed via side chain–side chain interactions mediate anion transport predominantly through the hydrophobic methylene CH_2_ groups that decorate the pore interior. The corresponding chloride transport current likely is very low (earlier pore-forming molecules all end up with <0.1 pA current [43,44,45,46]). Recording these low current signals requires advanced set-up, which we do not have in our current lab. Since TA molecules lack suitable functional groups to serve as the carrier for anion transport, we believe they should function as channels, particularly in view of recently reported pore-forming systems that generate pores through side chain–side chain interactions [43,44,45,46].

## 3. Materials and Methods

### 3.1. General Considerations

All the reagents were obtained from commercial suppliers and used as received unless otherwise noted. ^1^H and ^13^C NMR spectra were recorded on a Bruker AVANCE III HD 400 spectrometer (Bruker, Switzerland, German). Mass spectra were acquired with a Shimadzu LCMS-8030 (Shimadzu, Tokyo, Japan). The model number of the fluorescence spectrophotometer is RF-6000 (Shimadzu, Tokyo, Japan).

### 3.2. General Procedure for the Synthesis of TAn Molecules as Typified Using TA6

Trimesoyl chloride (265 mg, 1.00 mmol) and triethylamine (2.40 mL, 3.30 mmol) were dissolved in CH_2_Cl_2_ (20 mL), and then hexylamine (2.53 mL, 3.30 mmol) was added dropwise at 0 °C. The reaction mixture was stirred at room temperature for 48 h. The solvent was then removed in vacuo, and the crude product was purified by flash column chromatography (ethyl acetate/petroleum ether = 1:2, *v*:*v*) to afford the target compound **TA6** as a yellow solid. Yield: 360 mg, 80%. ^1^H NMR (400 MHz, chloroform-*d*) δ 8.28 (s, 3H), 6.74 (t, *J* = 5.7 Hz, 3H), 3.43 (q, *J* = 6.7 Hz, 7H), 1.61 (q, *J* = 7.2 Hz, 7H), 1.30 (qd, *J* = 10.4, 5.9 Hz, 18H), 0.89 (d, *J* = 6.5 Hz, 10H). ^13^C NMR (101 MHz, chloroform-*d*) δ 166.88, 135.41, 127.46, 40.29, 31.53, 29.38, 26.73, 22.62, 14.08. MS-ESI: calculated for [M + Na]^+^ (C_27_H_45_N_3_O_3_Na): *m*/*z* 482.35, found: *m*/*z* 482.41.

## 4. Conclusions

In summary, we have successfully established a class of structurally simple anion-transporting transporter system, which are easy to synthesize and displays excellent anion transport activities. As the best among the five transporter molecules studied, **TA6** demonstrates an extremely low *EC*_50_ value of 17.6 nM (0.022 mol% relative to lipids) for ClO_4_^−^, with selectivity factors 5–22-folds greater than four other types of anions (I^−^, NO_3_^−^, Br^−^ and Cl^−^). To our best knowledge, an *EC*_50_ value of 0.022 mol% is the lowest for ClO_4_^−^, with the second lowest being 0.052 mol%, as recently reported [43].

## Data Availability

Data are contained within the article.

## Supplementary Materials

The following supporting information can be downloaded at: https://www.mdpi.com/article/10.3390/molecules29051118/s1, indicating the ^1^H and ^13^C spectra of the new compounds, as well as the determination of *EC*_50_ value for **TA6**-mediated transport of anions. Figure S1: Determination of *EC_50_* values for Cl^−^ anions using the ratiometric values of I_460_/I_403_ at different concentrations as a function of time. Figure S2: Determination of *EC_50_* values for Br^−^ anions using the ratiometric values of I_460_/I_403_ at different concentrations as a function of time. Figure S3: Determination of *EC_50_* values for NO_3_^−^ anions using the ratiometric values of I_460_/I_403_ at different concentrations as a function of time. Figure S4: Determination of *EC_50_* values for I^−^ anions using the ratiometric values of I_460_/I_403_ at different concentrations as a function of time. Figure S5: Determination of *EC_50_* values for ClO_4_^−^ anions using the ratiometric values of I_460_/I_403_ at different concentrations as a function of time. Table S1: **TA**-mediated anion transport activities obtained using the HPTS assay. Scheme S1: Synthetic route that affords Trimesic amide-based pores.
