# Supplementary material for: An Exceptionally Active and Highly Selective Perchlorate Transporter Containing a Trimesic Amide Scaffold
Source: Molecules. 2024 Mar 1;29(5):1118. doi: 10.3390/molecules29051118 (PMC10935042; doi:10.3390/molecules29051118)
Supplement: Supplementary file 1 [file molecules-29-01118-s001.zip › molecules-2881221-supplementary.pdf]

An Exceptionally Active and Highly Selective Perchlorate Transporter Containing a Trimesic Amide Scaffold

Shaowen Deng 1, Zhongyan Li 2, Lin Yuan 1,* and Huaqiang Zeng 2,*

1 College of Chemistry and Bioengineering, Hunan University of Science and Engineering, Yongzhou 425100, China; shaowen_deng@163.com
2 College of Chemistry, Fuzhou University, Fuzhou 350116, China; lizhongyandongdong@126.com
** Correspondence: tcyl431102@163.com (L.Y.); hqzeng@fzu.edu.cn (H.Z.)*

1. **General Remarks** **S2**

2. **Synthetic Scheme and Structures of trimesic amides** **S3**

3. **Experimental Procedures and Compound Characterizations** **S4**

4. **Experimental Methods for Ion Transport Study** **S7**

5. **Determination of EC_{50} value for TA6-mediated transport of anions** **S13**

6. **$^{1}H/^{13}C$ NMR Spectra** **S15**

General Remarks

All the reagents were obtained from commercial suppliers and used as received unless otherwise noted. Aqueous solutions were prepared from ultrapure water. The organic solutions from all liquid extractions were dried over anhydrous Na_2SO_4 for a minimum of 15 min before filtration. Flash column chromatography was performed using pre-coated 0.2 mm silica plates from Qingdao Haiyang. Chemical yield refers to pure isolated substances. ^{1}H and ^{13}C NMR spectra were recorded on either a Bruker AVANCE III HD 400 spectrometer. The solvent signal of $CDCl_3$ was referenced at δ = 7.26 ppm. Coupling constants (*J* values) are reported in Hertz (Hz). ^{1}H NMR data are recorded in the order: chemical shift value, multiplicity (s, singlet; d, doublet; t, triplet; q, quartet; m, multiplet; br, broad), number of protons that gave rise to the signal and coupling constant, where applicable. ^{13}C spectra are proton-decoupled and recorded on Bruker AVANCE III HD 400 (Bruker, Switzerland, German). The solvent, $CDCl_3$, was referenced at δ = 77 ppm. $CDCl_3$ (99.8%-Deuterated) was purchased from Tenglong Weibo Technology and used without further purification. Mass spectra were acquired with Shimadzu LCMS-8030 (Shimadzu, Tokyo, Japan). The model number of the fluorescence spectrophotometer is RF-6000 (Shimadzu, Tokyo, Japan).

Scheme S1. Synthetic route that affords Trimesic amide-based pores

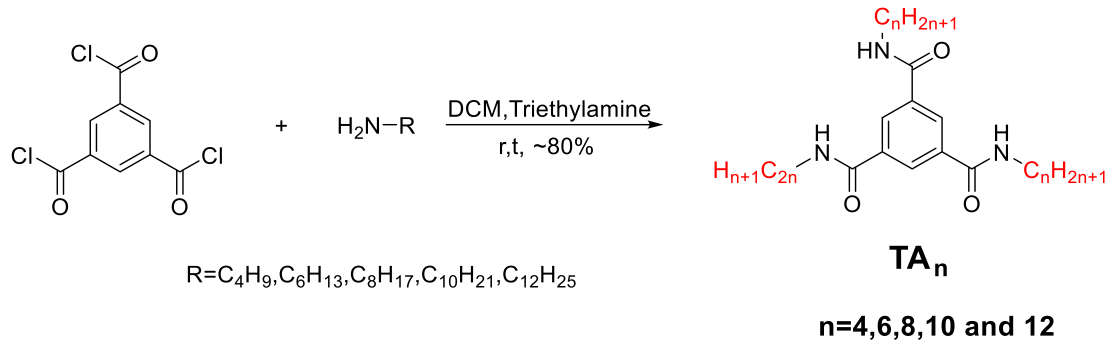

Experimental Procedures and Compound Characterizations

TA6

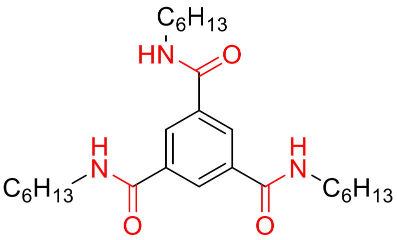

TA6

Take the synthesis of TA6 as an example. Trimesoyl chloride (265 mg, 1 mmol) and triethylamine (2.4 mL, 3.3 mmol) was dissolved in DCM (20 mL), then hexylamine (2.53 mL, 3.3 mmol) was added dropwise at 0 °C. The reaction mixture was stirred at room temperature for 48 h. The solvent was then was removed in vacuo, and the crude product was purified by flash column chromatography (Ethyl acetate: Petroleum ether = 1:2, v : v) to afford the target compound **TA6** as a yellow solid. Yield: 360 mg, 80%.^{1}H NMR (400 MHz, Chloroform-*d*) δ 8.28 (s, 3H), 6.74 (t, *J* = 5.7 Hz, 3H), 3.43 (q, *J* = 6.7 Hz, 7H), 1.61 (q, *J* = 7.2 Hz, 7H), 1.30 (qd, *J* = 10.4, 5.9 Hz, 18H), 0.89 (d, *J* = 6.5 Hz, 10H). ^{13}C NMR (101 MHz, Chloroform-*d*) δ 166.88, 135.41, 127.46, 40.29, 31.53, 29.38, 26.73, 22.62, 14.08. MS-ESI: calculated for $[M+Na]^+$ ($C_{27}H_{45}N_3O_3Na$)： m/z 482.35, found: m/z 482.41.

Preparation of TA4, TA8, TA10 and TA12 follows the same synthetic procedure as TA6.

TA4

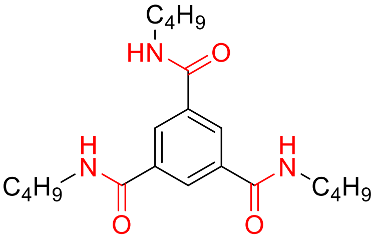

TA4

^{1}H NMR (400 MHz, Chloroform-*d*) δ 7.93 (s, 3H), 7.11 (d, *J* = 5.8 Hz, 3H), 3.41 (q, *J* = 6.7 Hz, 6H), 1.59 (p, *J* = 7.3 Hz, 6H), 1.40 (q, *J* = 7.4 Hz, 6H), 0.95 (t, *J* = 7.3 Hz, 9H). ^{13}C NMR (101 MHz, Chloroform-*d*) δ 165.90, 135.27, 127.97, 40.09, 31.55, 20.15, 13.77. MS-ESI: calculated for $[M+Na]^+$ ($C_{27}H_{45}N_3O_3Na$)： m/z 398.25, found: m/z 398.33.

TA8

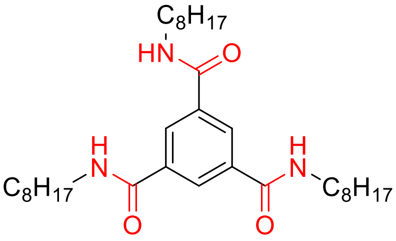

TA8

^{1}H NMR (400 MHz, Chloroform-*d*) δ 8.26 (s, 3H), 6.75 (s, 3H), 3.50 – 3.38 (m, 6H), 1.66 – 1.52 (m, 6H), 1.34 (s, 10H), 1.29 (d, *J* = 9.9 Hz, 20H), 0.87 (d, *J* = 7.2 Hz, 9H). ^{13}C NMR (101 MHz, Chloroform-*d*) δ 166.03, 135.19, 128.03, 40.45, 31.83, 29.51, 29.30, 29.24, 27.04, 22.67, 14.12. MS-ESI: calculated for $[M+Na]^+$ ($C_{27}H_{45}N_3O_3Na$)： m/z 566.44, found: m/z 566.52.

TA10

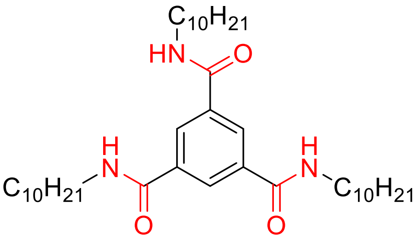

TA10

^{1}H NMR (400 MHz, Chloroform-*d*) δ 8.17 (d, *J* = 7.1 Hz, 3H), 6.95 (s, 3H), 3.42 (d, *J* = 9.4 Hz, 6H), 1.65 – 1.57 (m, 6H), 1.30 (d, *J* = 24.5 Hz, 42H), 0.87 (q, *J* = 7.5, 7.0 Hz, 9H).^{13}C NMR (101 MHz, Chloroform-*d*) δ 166.12, 135.20, 127.99, 40.45, 31.91, 29.61, 29.51, 29.37, 29.35, 27.07, 22.71, 14.14. MS-ESI: calculated for $[M+Na]^+$ ($C_{27}H_{45}N_3O_3Na$)： m/z 650.53, found: m/z 650.59.

TA12

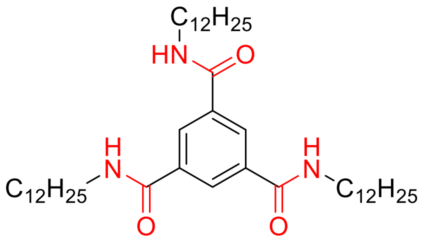

TA12

^{1}H NMR (400 MHz, Chloroform-*d*) δ 8.25 (s, 3H), 6.79 (s, 3H), 3.48 – 3.41 (m, 6H), 1.42 – 1.38 (m, 6H), 1.28 (d, J = 11.9 Hz, 54H), 0.88 (t, J = 6.7 Hz, 9H). ^{13}C NMR (101 MHz, Chloroform-*d*) δ 166.13, 135.23, 127.96, 40.43, 31.94, 29.70, 29.67, 29.61, 29.52, 29.39, 27.07, 22.71, 14.15. MS-ESI: calculated for $[M+Na]^+$ ($C_{27}H_{45}N_3O_3Na$)： m/z 734.63, found: m/z 734.72.

Experimental methods for ion transport study

Ion transport study using the HPTS assay and EC_{50} measurements using the Hill analysis. Egg yolk L-α-phosphatidylcholine (EYPC, 1 mL, 25 mg/mL in $CHCl_3$, Avanti Polar Lipids, USA) was added into a round-bottom flask. The solvent was removed under reduced pressure at 30 °C. After drying the resulting film under high vacuum overnight, the film was hydrated with the HEPES (4-(2-hydroxyethyl)-1-piperazine-ethane sulfonic acid) buffer solution (1 mL, 10 mM HEPES, 100 mM NaCl, pH = 7.0) containing pH sensitive dye HPTS (8-hydrox-ypyrene-1,3,6-trisulfonic acid, 1 mM) at room temperature for 60 min to give a milky suspension. The mixture was then subjected to 10 freeze-thaw cycles: freezing in liquid N_2 for 1 min and heating in 55 °C water bath for 2 min. The vesicle suspension was extruded through polycarbonate membrane (0.1 μm) to produce a homogeneous suspension of large unilamellar vesicles (LUVs) of about 120 nm in diameter with the HPTS encapsulated inside. The unencapsulated HPTS dye was separated from the LUVs by using size exclusion chromatography (stationary phase: Sephadex G-50, GE Healthcare, USA, mobile phase: HEPES buffer with 100 mM NaCl) and diluted with the mobile phase to yield 5 mL of 6.5 mM lipid stock solution.

This HPTS-containing LUV suspension (25 μL, 2.5 mM) was added to a HEPES buffer solution (1.95 mL, 10 mM HEPES, 100 mM NaCl at pH = 8.0) to create a pH gradient for ion transport study. A solution of channel molecules in DMSO was then injected into the suspension under gentle stirring. Upon the addition of channels, the emission of HPTS was immediately monitored at 510 nm with excitations at both 460 and 403 nm recorded simultaneously for 300 s using fluorescence spectrophotometer (SHIMADZU, Model RF-6000, Japan). At t = 300 s, an aqueous solution of Triton X-100 (20 μL, 20% v/v) was immediately added to achieve the maximum change in dye fluorescence emission intensity. After subtracting background intensity at t = 0, the final transport trace was obtained as a ratiometric value of I_{460}/I_{403} and normalized based on the ratiometric value of I_{460}/I_{403} after addition of triton at t = 300 s. The fractional changes R_{Cl^-} was calculated for each curve using the normalized value of I_{460}/I_{403} at t = 300 s before the addition of triton, with ratiometric value of I_{460}/I_{403} at t = 0 s as 0% and that of I_{460}/I_{403} at t = 300 s (obtained

after addition of triton) as 100%. Fitting the fractional transmembrane activity R_{Cl^-} vs channel concentration using the Hill equation: $Y=1/(1+(EC_{50}/[C])^n)$ gave the Hill coefficient n and EC_{50} values.

Sulphate-containing HPTS assay for cation selectivity. Egg yolk L-α-phosphatidylcholine (EYPC, 1 ml, 25 mg/mL in $CHCl_3$, Avanti Polar Lipids, USA). solvents were removed under reduced pressure at room temperature. After drying the resulting film under high vacuum overnight at room temperature, the film was hydrated with 4-(2- hydroxyethyl)-1-piperazine-ethane sulfonic acid (HEPES) buffer solution (1.0 mL, 10 mM HEPES, pH = 7.0) containing a pH sensitive dye 8-hydrox-ypyrene-1,3,6-trisulfonic acid (HPTS, 1 mM) in thermostatic shaker-incubator at room temperature for 60 min to give a milky suspension. The mixture was then subjected to 10 freeze-thaw cycles: freezing in liquid N_2 for 1 min and heating at 55 ^oC for 2 min. The vesicle suspension was extruded through polycarbonate membrane (0.1 μm) to produce a homogeneous suspension of large unilamellar vesicles (LUVs) of about 120 nm in diameter with HPTS encapsulated inside. The unencapsulated HPTS dye was separated from the LUVs by using size exclusion chromatography (stationary phase: Sephadex G-50, GE Healthcare, USA, mobile phase: HEPES buffer with pH = 7.0) and diluted with the mobile phase to yield 5 mL of 6.5 mM lipid stock solution.

The HPTS-containing LUV suspension (25 μL, 2.5 mM in 10 mM HEPES buffer, pH = 7.0) was added to a HEPES buffer solution (1.95 mL, 10 mM HEPES, 200 mM MCl at pH = 7.0, where $M^+ = Li^+$, Na^+, K^+, Rb^+ and Cs^+) for ion transport study. A solution of channel molecule TA6 at a final concentration of 0.6 μM in DMSO was then injected into the suspension under gentle stirring. Upon the addition of channels, the emission of HPTS was immediately monitored at 510 nm with excitations at both 460 and 403 nm recorded simultaneously for 300 s using fluorescence spectrophotometer (SHIMADZU, Model RF-6000, Japan) after which time an aqueous solution of Triton X-100 (20 μL, 20% v/v) was immediately added to achieve the maximum change in dye fluorescence emission. The final transport trace was obtained as a ratiometric value of I_{460}/I_{403} and normalized based on the ratiometric value of I_{460}/I_{403} after addition of triton.

Chloride transport using the SPQ Assay. Egg yolk L-α-phosphatidylcholine (EYPC, 1 ml,

25 mg/mL in $CHCl_3$, Avanti Polar Lipids, USA) was mixed in a roundbottom flask. The mixed solvents were removed under reduced pressure at 30 °C. After drying the resulting film under high vacuum overnight at room temperature, the film was hydrated $NaNO_3$ solution (1 mL, 200 mM) containing a Cl^- -sensitive dye 6-methoxy-N-(3- sulfopropyl)quinolinium (SPQ) (0.5 mM) in thermostatic shaker-incubator at room temperature for 60 min to give a milky suspension. The mixture was then subjected to 10 freeze-thaw cycles: freezing in liquid N_2 for 1 min and heating at 55 °C in water bath for 2 min. The vesicle suspension was extruded through polycarbonate membrane (0.1 μm) to produce a homogeneous suspension of large unilamellar vesicles (LUVs) of about 120 nm in diameter with SPQ encapsulated inside. The unencapsulated HPTS dye was separated from the LUVs by using size exclusion chromatography (stationary phase: Sephadex G-50, GE Healthcare, USA, mobile phase: 200 mM $NaNO_3$) and diluted with the mobile phase to yield 5 mL of 6.5 mM lipid stock solution.

The SPQ-containing LUV suspension (25 μL, 2.5 mM in 200 mM $NaNO_3$) was added to a NaCl solution (1.95 mL, 200 mM) to create an extravesicular chloride gradient. A solution of tripeptide molecule **TA6** in Acetonitrile at different concentrations was then injected into the suspension under gentle stirring. Upon the addition of pore-forming monopeptide molecules, the emission of SPQ was immediately monitored at 430 nm with excitations at 360 nm for 300 s using fluorescence spectrophotometer (SHIMADZU, Model RF-6000, Japan) after which time an aqueous solution of Triton X-100 (20 μL, 20% v/v) was immediately added to completely destroy the chloride gradient. The final transport trace was obtained by normalizing the fluorescence intensity using the equation of $I_f = [(I_t - I_1)/(I_0 - I_1)]$ where, I_f = Fractional emission intensity, I_t = Fluorescence intensity at time t, I_1 = Fluorescence intensity after addition of Triton X-100, and I_0 = Initial fluorescence intensity .

Membrane leaking and pore size determination using CF dye. Egg yolk L-α-phosphatidylcholine (EYPC, 1 ml, 25 mg/mL in $CHCl_3$, Avanti Polar Lipids, USA) was mixed in a round-bottom flask. The mixed solvents were removed under reduced pressure at 30 °C. After drying the resulting film under high vacuum overnight at room temperature, the film was hydrated with HEPES buffer solution (1 mL, 10 mM HEPES, 100 mM NaCl, pH = 7.5) containing a 5(6)-

fluorescein (CF, 50 mM) at room temperature for 60 minutes to give a milky suspension. The mixture was then subjected to 10 freeze-thaw cycles: freezing in liquid N_2 for 1 min and heating at 55 ^oC in water bath for 2 min. The vesicle suspension was extruded through polycarbonate membrane (0.1 μm) to produce a homogeneous suspension of large unilamellar vesicles (LUVs) of about 120 nm in diameter with CF encapsulated inside. The free unencapsulated CF dye was separated from the LUVs by using size exclusion chromatography (stationary phase: Sephadex G-50, GE Healthcare, USA, mobile phase: HEPES buffer with 100 mM NaCl) and diluted with the mobile phase to yield 5 mL of 6.5 mM lipid stock solution.

The CF-containing LUV suspension (25 μL, 2.5 mM in 10 mM HEPES buffer containing 100 mM NaCl at pH = 7.5) was added to a HEPES buffer solution (1.95 mL, 10 mM HEPES, 100 mM NaCl at pH = 7.5) to create a concentration gradient for CF dye transport study. A solution of **TA6** (2.5 μM) or natural poreforming peptide Melittin in DMSO at different concentrations was then injected into the suspension under gentle stirring. Upon the addition of pore-forming monopeptide molecules, the emission of CF was immediately monitored at 517 nm with excitations at 492 nm for 300 s using fluorescence spectrophotometer (SHIMADZU, Model RF-6000, Japan) after which time an aqueous solution of Triton X-100 (20 μL, 20% v/v) was immediately added to completely destroy the chloride gradient. The final transport trace was obtained by normalizing the fluorescence intensity using the equation of $I_f = [(I_t- I_0)/(I_l- I_0)]$ where, If = Fractional emission intensity, I_t = Fluorescence intensity at time t, I_l = Fluorescence intensity after addition of Triton X-100, and I_0 = Initial fluorescence intensity .

The HPTS assay in the presence of FCCP (carbonyl cyanide-4-(trifluoromethoxy) phenylhydrazone). The HPTS-containing LUV suspension (25 μL, 2.5 mM in 10 mM HEPES buffer containing 100 mM NaCl at pH = 7.0) was added to a HEPES buffer solution (1.93 mL, 10 mM HEPES, 100 mM NaCl) to create a pH gradient for ion transport study. A solution of FCCP (0.1 μM) and TA6 (0.35 μM) in DMSO was then injected into the suspension under gentle stirring at 20 s and 70 s, respectively. Upon the addition of poreforming monopeptide molecules, the emission of HPTS was immediately monitored at 510 nm with excitations at both 460 and 403 nm recorded simultaneously for 300 s using fluorescence spectrophotometer (SHIMADZU, Model RF-

6000, Japan). 300 s later, aqueous solution of Triton X-100 (20 μL, 20% v/v) was immediately added to achieve the maximum change in dye fluorescence emission. The final transport trace was obtained as a ratiometric value of I_{460}/I_{403} and normalized based on the ratiometric value of I_{460}/I_{403} after addition of triton.

The HPTS assay for anion selectivity. The HPTS-containing LUV suspension (25 μL, 2.5 mM in 10 mM HEPES buffer containing 100 mM NaX where X^- = Cl^- , Br^- , I^- , NO_3^- and ClO_4^- at pH = 7.0) was added to a HEPES buffer solution (1.95 mL, 10 mM HEPES, 100 mM NaX, where X^- = Cl^- , Br^- , I^- , NO_3^- and ClO_4^- at pH= 8.0) to create a pH gradient for ion transport study. A solution of peptides at specified concentrations in DMSO was then injected into the LUV suspension under gentle stirring. Upon the addition of pore-forming monopeptide molecules, the emission of HPTS was immediately monitored at 510 nm with excitations at both 460 and 403 nm recorded simultaneously for 300 s using fluorescence spectrophotometer (SHIMADZU, Model RF-6000, Japan) after which time an aqueous solution of Triton X-100 (20 μL, 20% v/v) was immediately added to achieve the maximum change in dye fluorescence emission. The final transport trace was obtained as a ratiometric value of I_{460}/I_{403} and normalized based on the ratiometric value of I_{460}/I_{403} after addition of triton.

Sulphate-containing HPTS assay for cation selectivity. Egg yolk L-α- phosphatidylcholine (EYPC, 1 ml, 25 mg/mL in $CHCl_3$, Avanti Polar Lipids, USA) was added into a round-bottom flask. The solvent was removed under reduced pressure at room temperature. After drying the resulting film under high vacuum overnight at 30 °C. After drying the resulting film under high vacuum overnight, the film was hydrated with 4-(2- hydroxyethyl)-1-piperazine-ethane sulfonic acid (HEPES) buffer solution (1.0 mL, 10 mM HEPES, pH = 7.0) containing a pH sensitive dye 8-hydrox-ypyrene-1,3,6-trisulfonic acid (HPTS, 1 mM) in thermostatic shaker-incubator at room temperature for 60 min to give a milky suspension. The mixture was then subjected to 10 freeze-thaw cycles: freezing in liquid N_2 for 1 min and heating at 55 °C for 2 min. The vesicle suspension was extruded through polycarbonate membrane (0.1 μm) to produce a homogeneous suspension of large unilamellar vesicles (LUVs) of about 100 nm in diameter with HPTS encapsulated inside. The

unencapsulated HPTS dye was separated from the LUVs by using size exclusion chromatography (stationary phase: Sephadex G-50, GE Healthcare, USA, mobile phase: HEPES buffer with , pH = 7.0) and diluted with the mobile phase to yield 5 mL of 6.5 mM lipid stock solution.

The HPTS-containing LUV suspension (25 μL, 2.5 mM in 10 mM HEPES buffer, pH = 7.0) was added to a HEPES buffer solution (1.95 mL, 10 mM HEPES, 200 mM M_2SO_4 at pH = 7.0, where M^+ = Na^+ , K^+) for ion transport study. A solution of channel molecule **TA6** at a final concentration of 2.5 μM (or gramicidin A at 2 μM) in DMSO was then injected into the suspension under gentle stirring. Upon the addition of channels, the emission of HPTS was immediately monitored at 510 nm with excitations at both 460 and 403 nm recorded simultaneously for 300 s using fluorescence spectrophotometer (SHIMADZU, Model RF-6000, Japan) after which time an aqueous solution of Triton X-100 (20 μL, 20% v/v) was immediately added to achieve the maximum change in dye fluorescence emission. The final transport trace was obtained as a ratiometric value of I_{460}/I_{403} and normalized based on the ratiometric value of I_{460}/I_{403} after addition of triton.

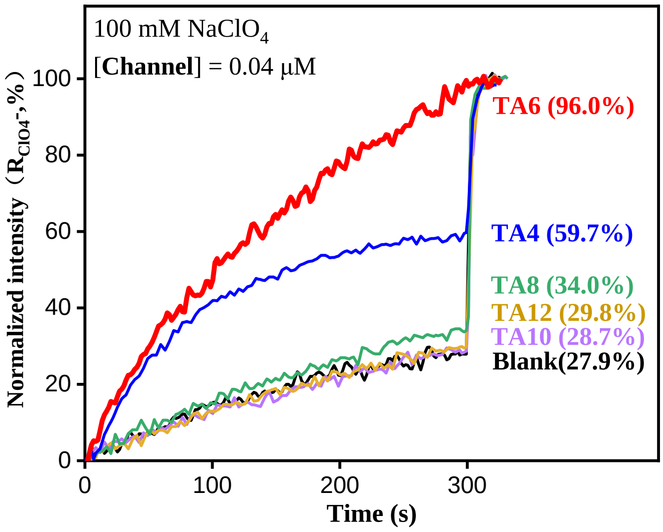

Table S1. TA-mediated anion transport activities obtained using the HPTS assay.

Anions tested	**TA6**	**TA4**	**TA8**	**TA12**	**TA10**
ClO_4^{-} [a]	94%	44%	8.5%	2.8%	1.1%
Cl^{-} [b]	97%	36%	15%	6.1%	4.1%

[a] Transporter concentration at 0.04 μM. [b] Transporter concentration at 2.5 μM.

Determination of EC_{50} value for TA6-mediated transport of anions

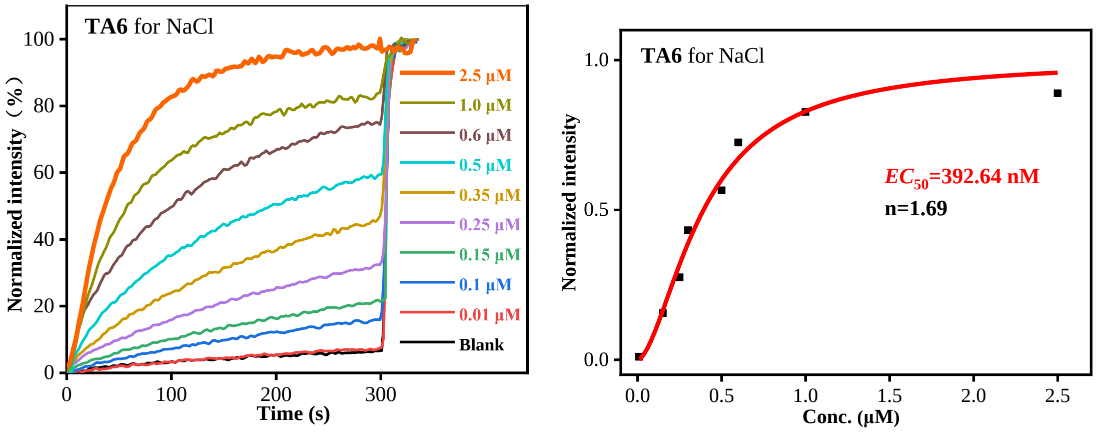

Figure S1 Determination of EC_{50} values for Cl^- anions using the ratiometric values of I_{460}/I_{403} at different concentrations as a function of time.

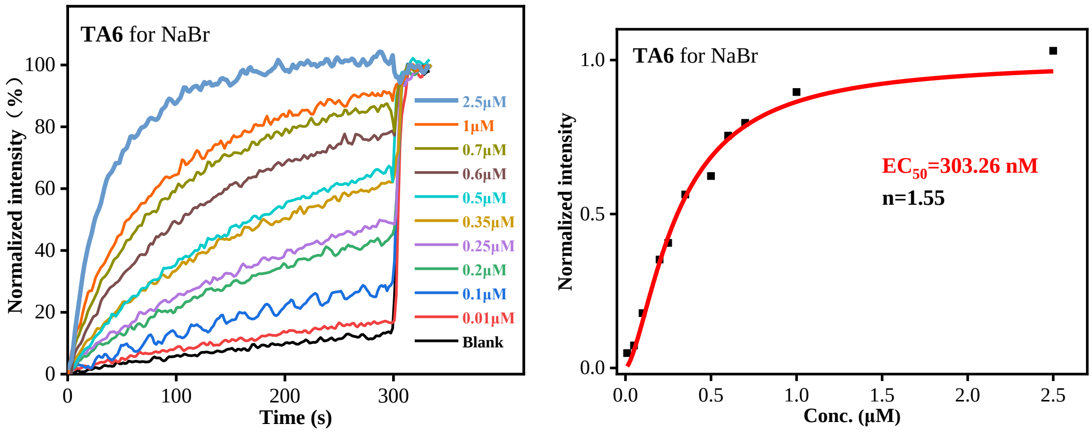

Figure S2 Determination of EC_{50} values for Br^- anions using the ratiometric values of I_{460}/I_{403} at different concentrations as a function of time.

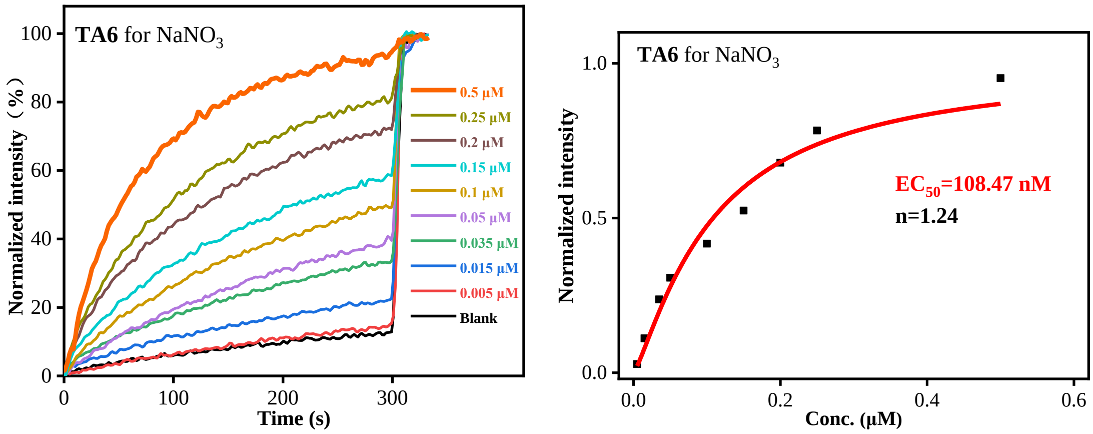

Figure S3. Determination of EC_{50} values for NO_3^- anions using the ratiometric values of I_{460}/I_{403} at different concentrations as a function of time.

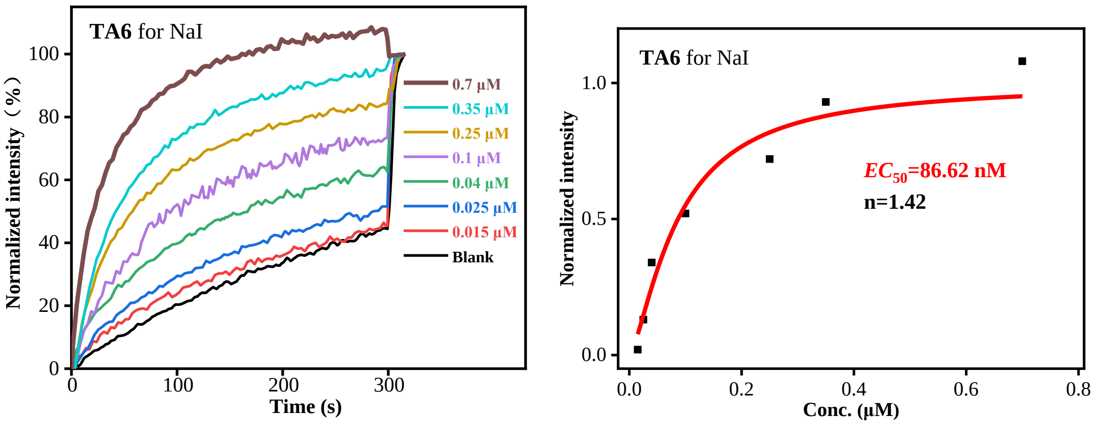

Figure S4 Determination of EC_{50} values for I^- anions using the ratiometric values of I_{460}/I_{403} at different concentrations as a function of time.

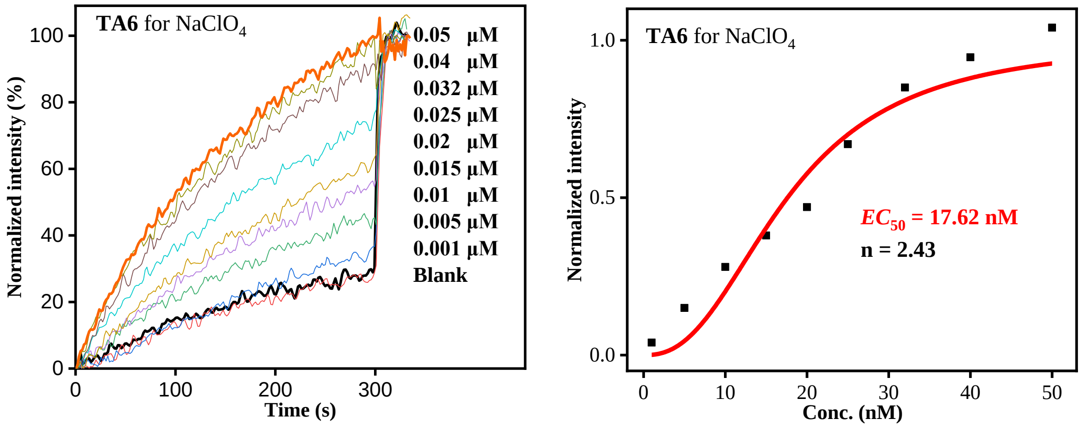

Figure S5 Determination of EC_{50} values for ClO_4^- anions using the ratiometric values of I_{460}/I_{403} at different concentrations as a function of time.

^1H/^{13}C NMR Spectra

TA4

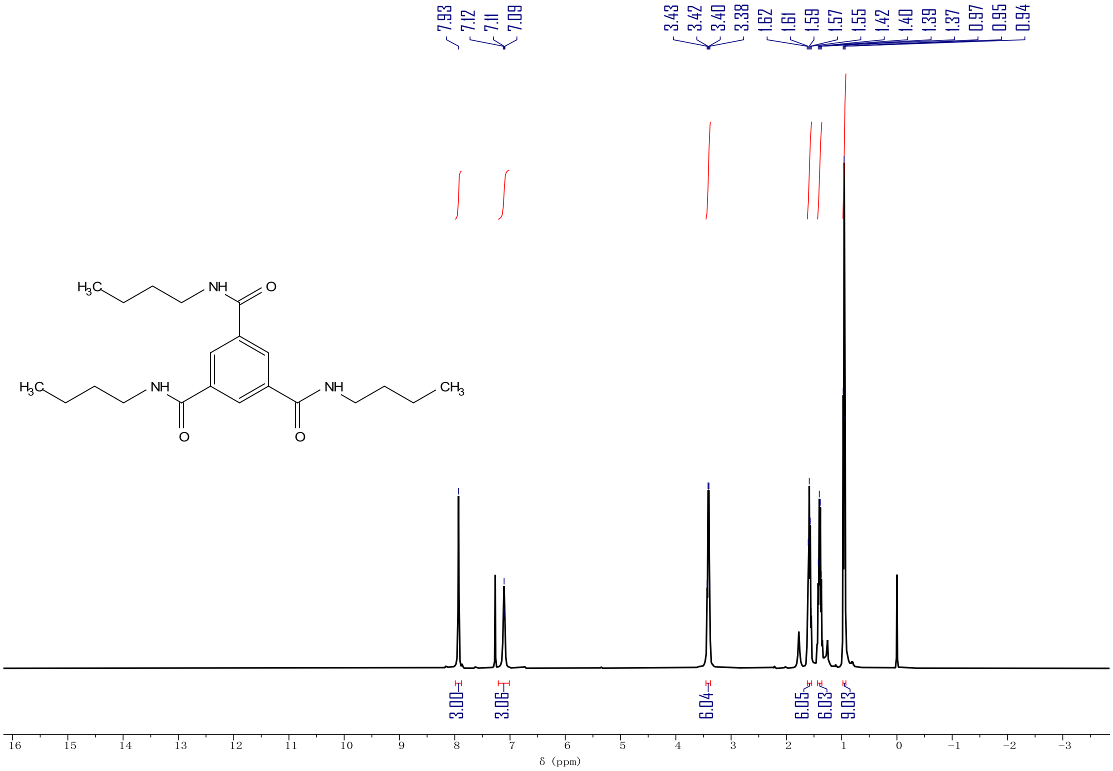

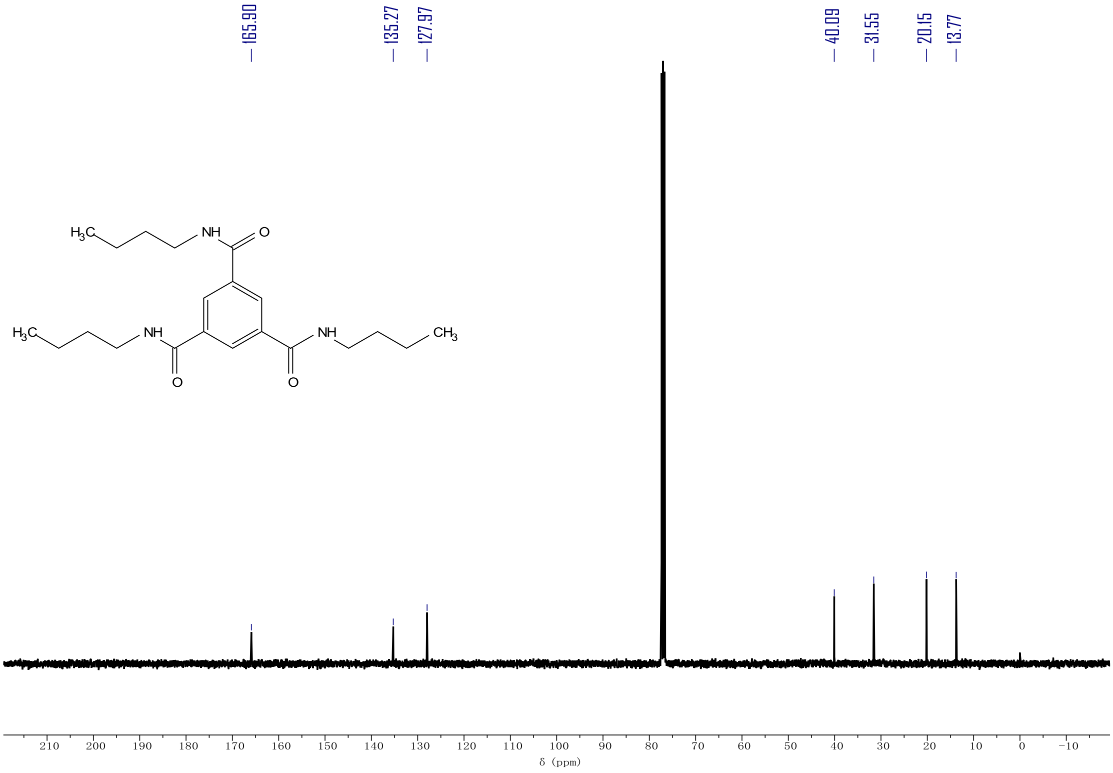

TA6

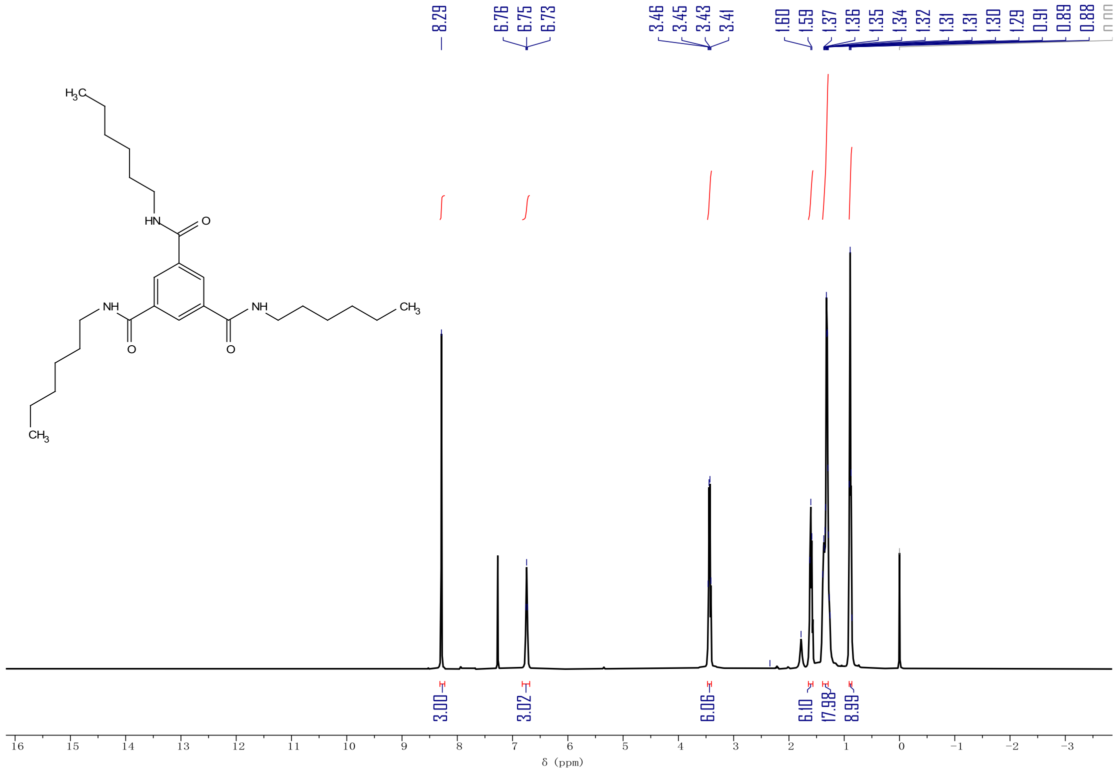
8.29
6.76
6.75
6.73
3.46
3.45
3.43
3.41
1.60
1.59
1.37
1.36
1.35
1.34
1.32
1.31
1.31
1.30
1.29
0.91
0.89
0.88
3.00
3.02
6.06
6.10
17.98
8.99
δ (ppm)

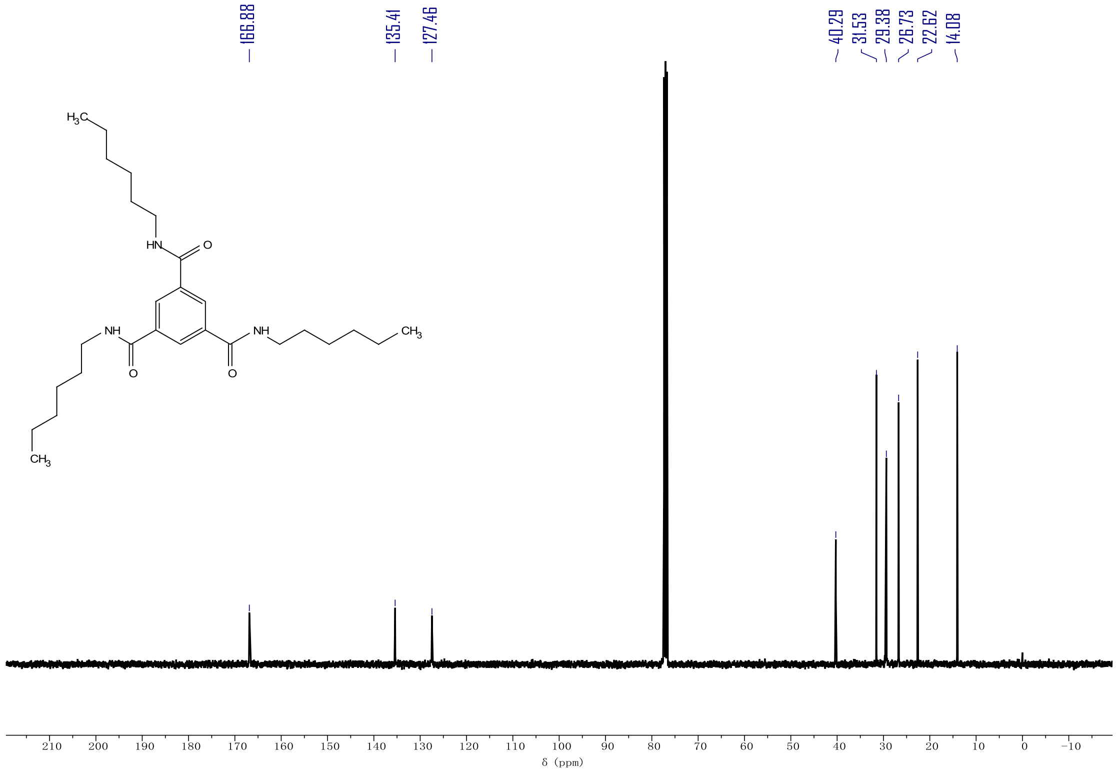
166.88
135.41
127.46
40.29
31.53
29.38
26.73
22.62
14.08
δ (ppm)

TA8

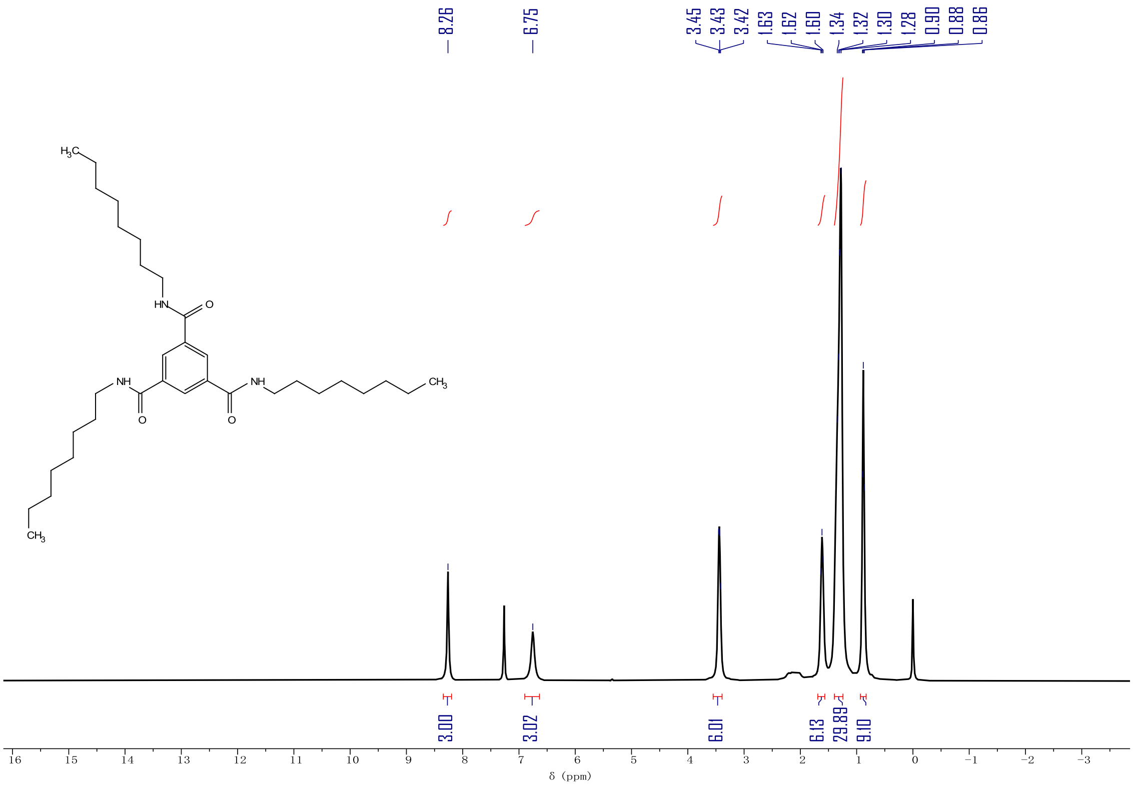

8.26
6.75
3.45
3.43
3.42
1.63
1.62
1.60
1.34
1.32
1.30
1.28
0.90
0.88
0.86
H3C
HN
O
NH
NH
CH3
O
O
CH3
3.00
3.02
6.01
6.13
29.89
9.10
δ (ppm)

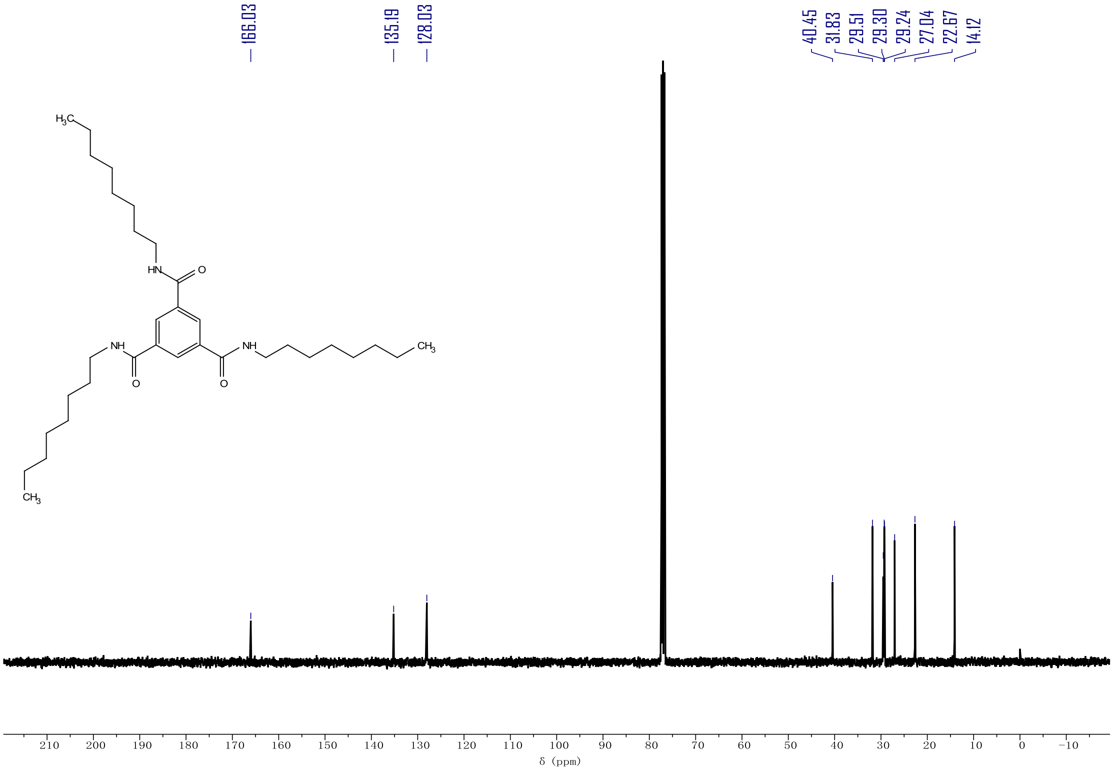

166.03
135.19
128.03
40.45
31.83
29.51
29.30
29.24
27.04
22.67
14.12
H3C
HN
O
NH
NH
CH3
O
O
CH3
δ (ppm)

TA10

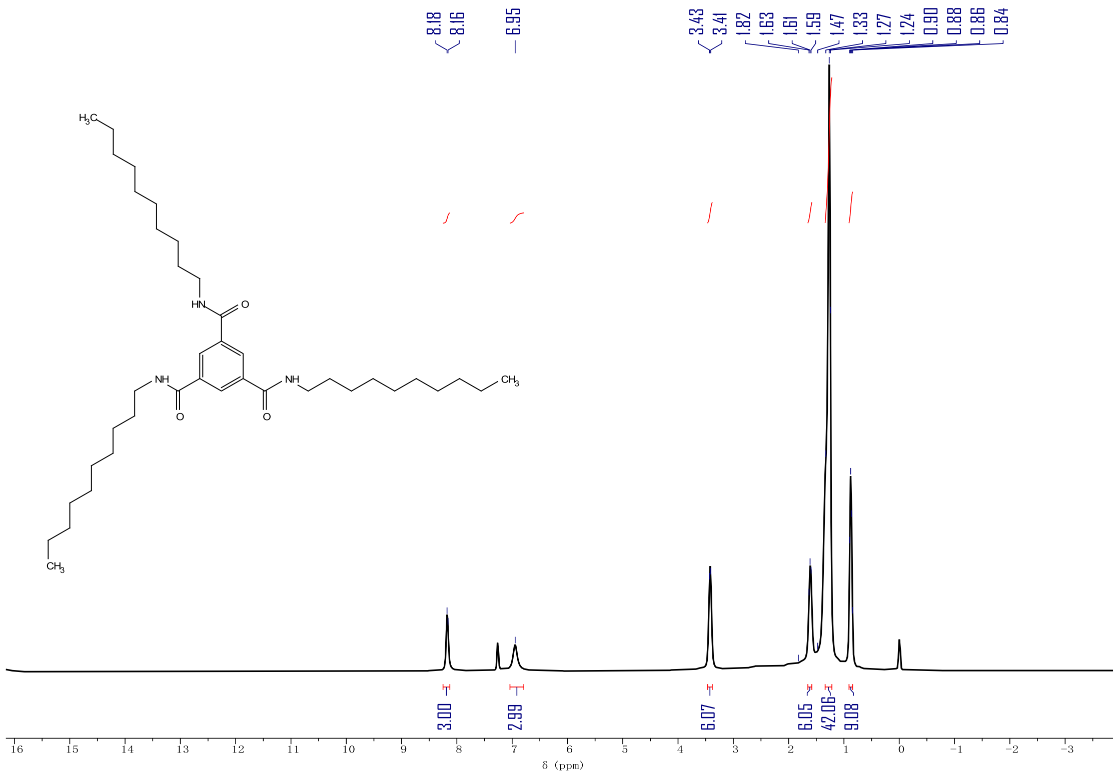
8.18
8.16
6.95
3.43
3.41
1.82
1.63
1.61
1.59
1.47
1.33
1.27
1.24
0.90
0.88
0.86
0.84
H3C
HN
O
NH
NH
CH3
O
O
CH3
3.00
2.99
6.07
6.05
42.06
9.08
δ (ppm)

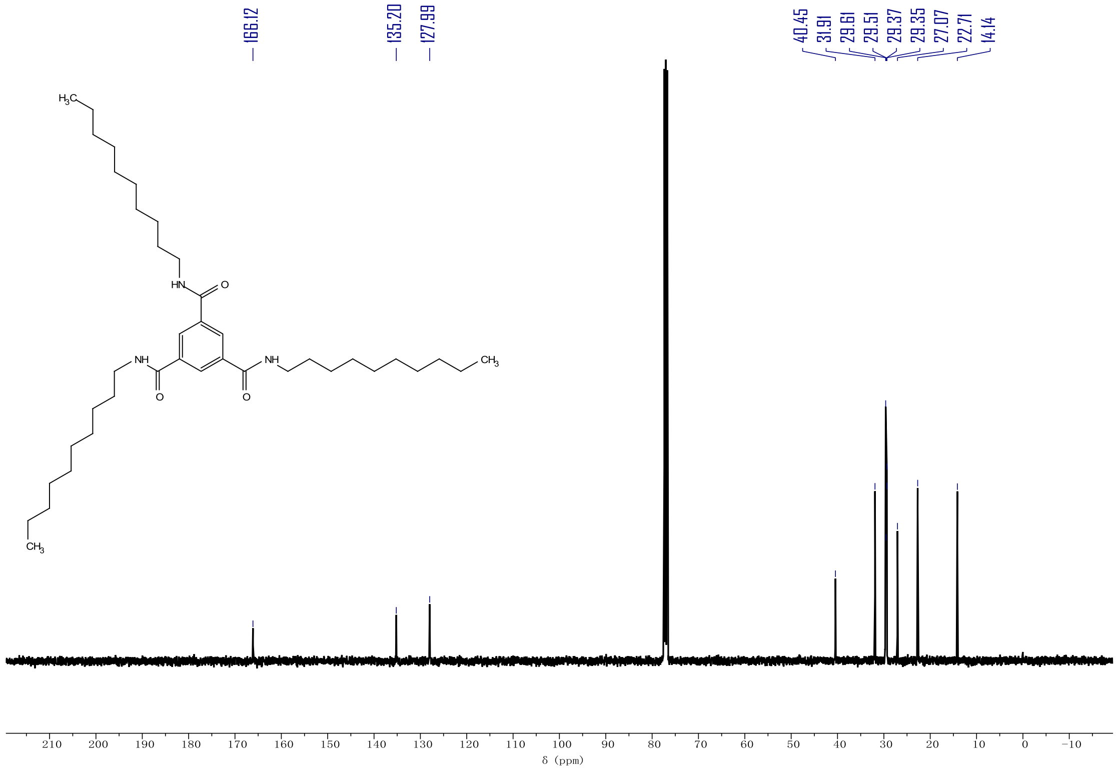
166.12
135.20
127.99
40.45
31.91
29.61
29.51
29.37
29.35
27.07
22.71
14.14
H3C
HN
O
NH
NH
CH3
O
O
CH3
δ (ppm)

TA12

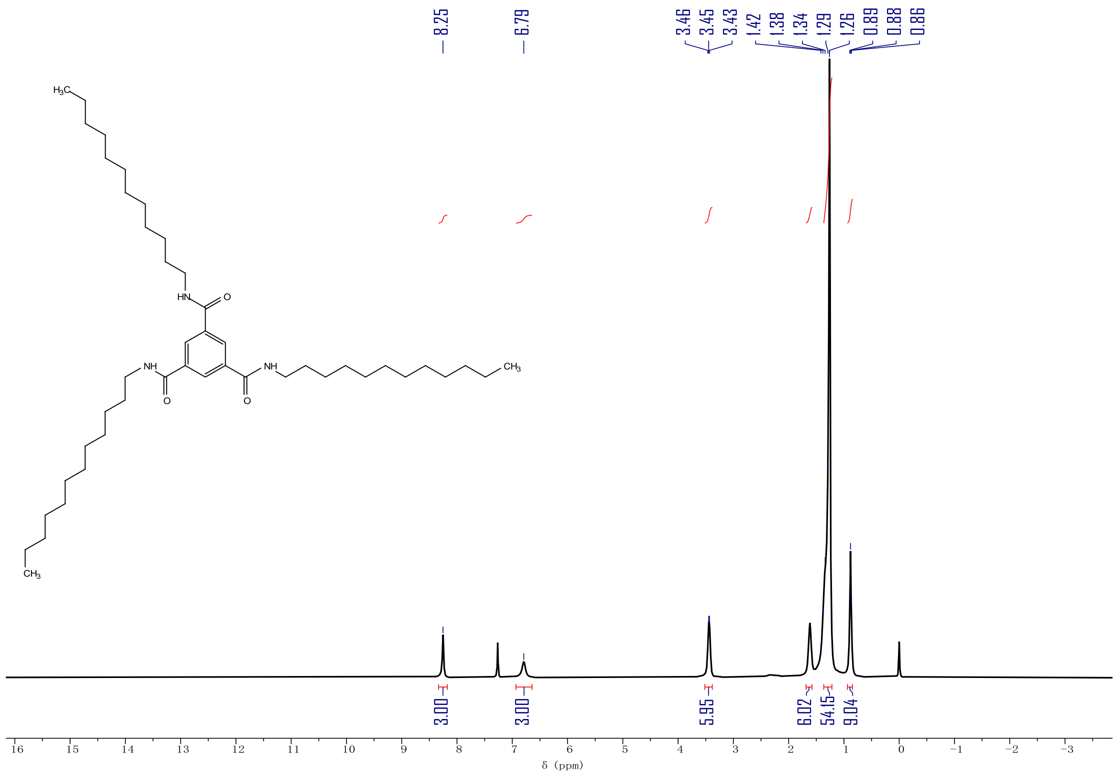
8.25
6.79
3.46
3.45
3.43
1.42
1.38
1.34
1.29
1.26
0.89
0.88
0.86
3.00
3.00
5.95
6.02
54.15
9.04
δ (ppm)

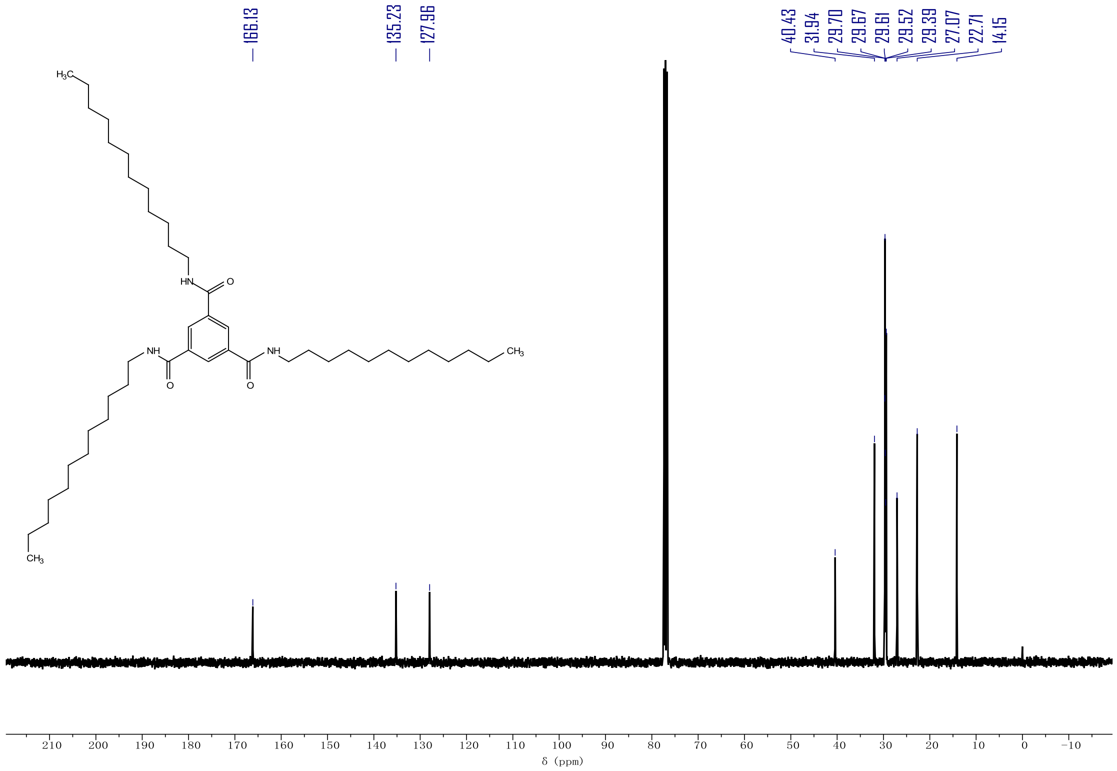
166.13
135.23
127.96
40.43
31.94
29.70
29.67
29.61
29.52
29.39
27.07
22.71
14.15
δ (ppm)
